# Clinical Characteristics and Outcomes of Pediatric Oncology Patients Admitted to the Pediatric Intensive Care Unit: A Single Center Experience in Saudi Arabia

**DOI:** 10.3390/children13010058

**Published:** 2025-12-31

**Authors:** Wafaa Aljizani, Fatmah Othman, Faisal Alrashed, Faisal Althaqeel, Obaid Alfuraydi

**Affiliations:** 1 Division of Pediatric Hematology/Oncology/SCT, King Abdullah Specialist Children Hospital, Riyadh 14611, Saudi Arabia; 2 King Abdullah International Medical Research Center, Riyadh 11481, Saudi Arabia; othmanf@ksau-hs.edu.sa; 3 College of Medicine, King Saud bin Abdulaziz University for Health Sciences, Riyadh 11481, Saudi Arabia; alrashed071@ksau-hs.edu.sa (F.A.); althaqeel074@ksau-hs.edu.sa (F.A.); alfredei190@ksau-hs.edu.sa (O.A.)

**Keywords:** pediatric oncology, pediatric intensive care unit (PICU), sepsis, organ dysfunction, mortality, mechanical ventilation

## Abstract

**Highlights:**

**What are the main findings?**

- Sepsis and respiratory failure were the leading reasons for PICU admission among pediatric oncology patients, with hematologic malignancies representing the majority of cases.
- Mechanical ventilation and prior therapeutic interventions were the strongest independent predictors of mortality, whereas PRISM-IV score was not associated with mortality in adjusted analysis.

**What are the implications of the main findings?**

- Early recognition of high-risk patients and rapid initiation of organ-supportive therapies are crucial to improving survival in pediatric oncology patients requiring intensive care.
- Reliance on general severity scoring tools may be insufficient; targeted risk-stratification strategies may be needed to better identify children at greatest risk of deterioration.

**Abstract:**

Background/Objectives: Advances in pediatric oncology have improved survival; however, critically ill children with cancer remain at high risk for adverse outcomes and frequently require admission to the pediatric intensive care unit (PICU). Despite the rising burden of pediatric cancer in Saudi Arabia, data on PICU utilization and outcomes remain limited. This study aimed to describe the clinical characteristics, critical care interventions, and outcomes of pediatric oncology patients admitted to a tertiary PICU and to identify predictors of mortality. Methods: This is a retrospective cohort study was conducted including pediatric oncology patients (<14 years) admitted to the PICU at King Abdullah Specialized Children’s Hospital, Riyadh, between 2015 and 2021. Demographic, oncologic, and clinical variables; admission indications; PRISM-IV scores; and PICU interventions were collected. Predictors of mortality were evaluated using Cox proportional hazards modeling. Results: A total of 126 pediatric oncology patients were admitted to the PICU during the study period. The median age was 6 years (IQR 3–11), and 59% were female. Hematologic malignancies accounted for 63% of admissions. Sepsis (41%) and respiratory failure (21%) were the leading indications for PICU admission. Comorbidities were present in 33% of patients, and 70% had received prior therapeutic interventions, most commonly chemotherapy. Organ dysfunction occurred in 39% of patients, including 32% with multiorgan failure. Mechanical ventilation was required in 35% of patients, vasopressor support in 30%, and dialysis in a smaller proportion. The overall mortality rate was 19%, with more than half of deaths occurring during the PICU stay. Non-survivors had higher rates of comorbidities and invasive organ support, and higher PRISM scores. Mechanical ventilation (HR 3.02; 95% CI 1.16–7.60) and prior therapeutic interventions (HR 3.19; 95% CI 1.24–8.19) were independent predictors of mortality. Conclusions: Pediatric oncology patients admitted to the PICU experience substantial morbidity and mortality, underscoring the need for early risk identification and optimized supportive care.

## 1. Introduction

The global burden of childhood cancer continues to rise. In 2022, the International Agency for Research on Cancer (IARC) estimated approximately 275,000 new cancer cases among children and adolescents aged 0–19 years worldwide [1]. In Saudi Arabia, the 2014 Saudi Cancer Registry reported 822 new pediatric cancer cases, and more recent national data indicate an incidence of 15.3 per 100,000 among individuals under 20 years of age [2]. Over recent decades, survival outcomes for children with cancer have markedly improved at the population level, with overall five-year survival increasing from approximately 40% in the 1970s to more than 80% in 2017 [3]. Despite these advances, substantial global disparities persist; more than 90% of children with cancer in high-income countries achieve long-term cure, compared with less than 30% in low- and middle-income countries [4].

Advances in chemotherapy, multimodal treatment approaches, and supportive care have significantly improved outcomes for pediatric oncology patients [5]. However, pediatric oncology treatment involves cytotoxic chemotherapy and intensive multimodal therapies that confer substantial toxicity and immunosuppression, predisposing affected children to severe complications and critical illness. Immunosuppression related to both the underlying malignancy and its treatment predisposes patients to severe infections, respiratory compromise, and organ dysfunction, frequently necessitating critical care support. Up to 38% of pediatric oncology patients require at least one admission to the pediatric intensive care unit (PICU) during their treatment course, most commonly within the first three years following diagnosis [5,6].

PICU admission in this population is typically precipitated by respiratory failure, sepsis, multiorgan dysfunction, or disease-specific complications [3,6,7] Hematologic malignancies are associated with a higher risk of critical illness due to greater degrees of immunosuppression and treatment intensity [4,6,7,8]. The need for invasive organ support, including mechanical ventilation and vasoactive therapy, has been consistently associated with worse outcomes and increased mortality [8,9,10]. In low- and middle-income countries, reported PICU mortality rates among pediatric oncology patients range from 27.8% to over 51% [9,10,11], whereas high-resource settings demonstrate substantially lower mortality. For example, a recent multicenter Italian cohort reported a mortality rate of 15%, suggesting improved outcomes within a high-income healthcare setting, while acknowledging potential variability in critical care resources across centers [10]. Severity-of-illness scoring systems, such as the Pediatric Risk of Mortality (PRISM) score, are widely used to assess physiological derangement at PICU admission and have been shown to correlate with observed mortality [12].

Despite the expansion of pediatric oncology services in Saudi Arabia and the increasing number of newly diagnosed childhood cancer cases, outcomes among pediatric oncology patients requiring PICU admission have not been systematically evaluated. Published data remain limited, and the burden of critical illness, patterns of PICU utilization, and predictors of mortality in this population are not well characterized at the national level.

To address this gap, we evaluated PICU admission rates, clinical characteristics, critical care interventions, and mortality outcomes among pediatric oncology patients admitted to a tertiary care PICU in Saudi Arabia between 2015 and 2021. Our objective was to delineate the burden of critical illness in this population and identify predictors of mortality to inform future improvements in supportive and critical care delivery.

## 2. Materials and Methods

### 2.1. Study Design and Setting

A retrospective cohort study was conducted in the Pediatric Intensive Care Unit (PICU) at King Abdullah Specialized Children’s Hospital (KASCH), Riyadh, Saudi Arabia. KASCH is a tertiary referral pediatric center within the National Guard Health Affairs system, serving approximately 1.15 million beneficiaries and providing advanced pediatric oncology and intensive care services. The PICU is a high-resource unit with access to comprehensive organ support and multidisciplinary subspecialty care.

Electronic medical records were reviewed using the BESTCare electronic health record system (Oracle Cerner, Kansas City, MO, USA) to extract clinical, demographic, and outcome data for all eligible patients admitted between January 2015 and December 2021. The study period corresponds to a phase of ongoing maturation and expansion of pediatric oncology and critical care services in Saudi Arabia, including the development of specialized tertiary referral centers and enhancements in supportive and intensive care capabilities, which may have influenced observed outcomes.

### 2.2. Study Population

Pediatric oncology patients aged <14 years admitted to the PICU during the study period were eligible for inclusion. This age cutoff reflects institutional policy and national healthcare practice in Saudi Arabia, where pediatric oncology and pediatric intensive care services are provided to patients younger than 14 years, while older adolescents are typically managed within adult oncology and intensive care services. Oncology patients were defined as those with a histopathologically confirmed diagnosis of a hematologic malignancy or solid tumor. Patients admitted solely for postoperative monitoring were excluded.

### 2.3. Data Collection and Study Variables

Demographic and clinical variables collected included age at PICU admission, sex, source of admission (oncology ward, emergency department, or other), presence of comorbidities, prior oncologic therapies, and the type of treatment received before PICU admission.

Underlying malignancies were classified according to the World Health Organization (WHO) classification of childhood cancers and categorized as hematologic malignancies or solid tumors. Hematologic malignancies included B-cell acute lymphoblastic leukemia (B-ALL), T-cell acute lymphoblastic leukemia (T-ALL), acute myeloid leukemia (AML), hemophagocytic lymphohistiocytosis (HLH), non-Hodgkin lymphoma (NHL), and acute lymphoblastic leukemia (ALL). Solid tumors included neuroblastoma, intracranial solid tumors such as medulloblastoma and astrocytoma, bone tumors including osteosarcoma and Ewing sarcoma, kidney tumors such as Wilms tumor, rhabdomyosarcoma, and other solid tumors.

PICU-related variables included the reason for admission; the requirement and duration of mechanical ventilation; the use of vasopressor support; and the need for renal replacement therapy. Severity of illness at PICU admission was assessed using the Pediatric Risk of Mortality IV (PRISM IV) score, calculated from the worst physiological values recorded during the first 24 h of PICU admission [12].

Organ dysfunction was defined as the presence of clinically significant dysfunction of one or more organ systems occurring during the PICU stay and requiring active medical intervention or organ support, including respiratory failure requiring mechanical ventilation, cardiovascular dysfunction requiring vasopressor support, renal dysfunction requiring renal replacement therapy, or documented multiorgan failure involving two or more organ systems.

### 2.4. Outcomes

The primary outcome measures of interest were mortality during PICU admission, mortality following PICU discharge, and length of PICU stay.

### 2.5. Statistical Analysis

Descriptive statistics were used to summarize demographic and clinical characteristics. Continuous variables were presented as means with standard deviations or medians with interquartile ranges (IQRs), as appropriate based on data distribution, which was assessed using the Shapiro–Wilk test. Categorical variables were summarized as frequencies and percentages.

Comparisons between groups (survivors vs. non-survivors and hematologic vs. solid malignancies) were performed using the chi-square test or Fisher’s exact test for categorical variables and the Mann–Whitney U test for continuous variables.

Univariate Cox proportional hazards regression analysis was conducted to identify predictors of PICU mortality. Variables with a *p*-value < 0.05 in univariate analysis were entered into a multivariable Cox proportional hazards model with clustered standard errors to estimate adjusted hazard ratios (HRs) and 95% confidence intervals (CIs). The proportional hazards assumption was assessed using log–log survival plots and the STATA “estat phtest” command.

Survival probability was estimated using Kaplan–Meier analysis. Statistical significance was defined as a two-sided *p*-value < 0.05. All statistical analyses were performed using Stata version 15.0 (StataCorp, College Station, TX, USA).

### 2.6. Ethical Approval

The study received approval from the Institutional Review Board of King Abdullah International Medical Research Center (KAIMRC) (Approval No.: RC18.334R). All patient data were de-identified to maintain confidentiality.

## 3. Results

### 3.1. Incidence and Characteristics of PICU Admission

King Abdullah Specialized Children’s Hospital is a large tertiary pediatric referral center that provides comprehensive subspecialty care, including pediatric oncology and intensive care services. During the study period (2015–2021), the PICU managed a high volume of critically ill pediatric patients annually. All pediatric oncology patients admitted to the PICU during this period were screened for eligibility. Of these, 126 patients aged <14 years with a confirmed diagnosis of malignancy met the inclusion criteria and were included in the final analysis, representing 42.1% of pediatric oncology patients at the institution during the study period. Of these, 100 patients (79%) were admitted once, while 26 patients (21%) required multiple admissions.

The median age at PICU admission was 6 years (IQR 3–11), and 59% of patients were female. Most PICU admissions were transfers from the oncology ward, whereas 26.1% were transferred from the emergency department. Patient demographic and clinical characteristics are summarized in Table 1.

Approximately one-third (33%) of patients had preexisting comorbidities, most commonly genetic disorders (23%). Among patients who received oncologic treatment prior to PICU admission (*n* = 89), chemotherapy alone was the most common modality, administered to 64 patients (71.9%). Combination therapies included chemotherapy with radiotherapy in 7 patients (7.8%), chemotherapy with radiotherapy and surgery in 5 patients (5.6%), and chemotherapy with surgery in 4 patients (4.4%). A smaller proportion received chemotherapy combined with targeted therapy (3.3%) or steroids alone (2.2%). Other treatment modalities accounted for 4.4% of cases. These findings highlight that the majority of PICU admissions occurred in the context of recent exposure to cytotoxic therapy.

**Figure 1:**
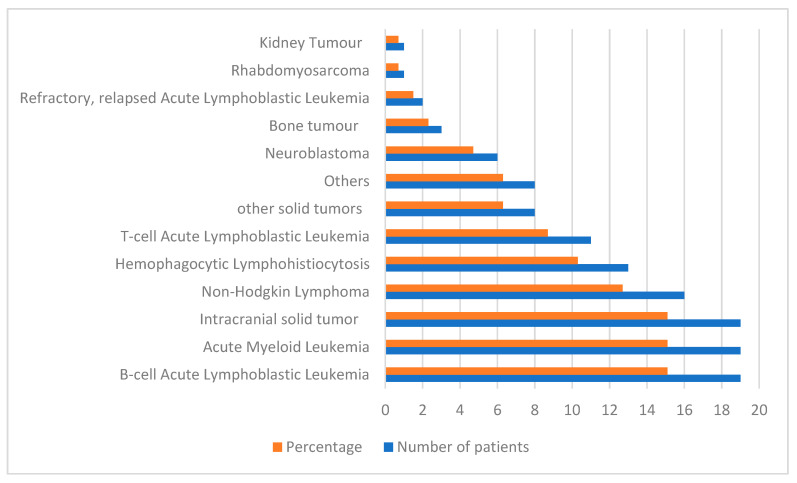
Distribution of underlying malignancies among pediatric oncology patients admitted to the pediatric intensive care unit (PICU). The “Others” category includes rare hematologic malignancies, such as Hodgkin lymphoma, non-Langerhans-cell histiocytosis, and marginal zone lymphoma. The “other solid tumors” category includes extracranial germ cell tumors, embryonal carcinoma, adrenocortical carcinoma, and pleuropulmonary carcinoma.

The leading indications for PICU admission were sepsis (41%) and respiratory failure (21%). Hematologic malignancies accounted for 63% of all admissions (Figure 1), with B-cell acute lymphoblastic leukemia (B-ALL) representing 15% of PICU admissions.

### 3.2. PICU Interventions and Organ Dysfunction

During PICU admission, 50 patients (39%) developed organ failure. Among these, 34 patients (68%) experienced single-organ failure, while 16 patients (32%) developed multi-organ failure involving two or more organ systems. The remaining 76 patients (61%) did not meet criteria for organ failure as defined by the need for organ-supportive interventions. Regarding intensive care interventions, mechanical ventilation was required in 35% of patients, and vasopressor support was administered to 30%. Renal replacement therapy was required in 2 patients (1.6%), with a median duration of dialysis of 3 days. Details of intensive care interventions and outcomes are summarized in Table 2.

### 3.3. Study Outcomes

The overall mortality rate among the study population was 19% (25/126 patients). Among these, 13 deaths (52%) occurred during the PICU admission, while 12 deaths (48%) occurred after discharge from the PICU.

During the PICU stay, causes of death included sepsis (*n* = 10), of which five progressed to multiorgan failure, respiratory failure (*n* = 2), and disease progression (*n* = 1).

Four additional deaths occurred after transfer from the PICU but during the same hospital admission and were attributed to gastrointestinal bleeding (*n* = 1), sepsis leading to multi-organ failure (*n* = 1), respiratory failure (*n* = 1), and disease progression (*n* = 1).

The remaining eight deaths occurred after hospital discharge during subsequent admissions and were related to sepsis (*n* = 4), disease progression (*n* = 2), and respiratory failure (*n* = 2) Table 3.

Compared with patients who survived, those who died had a higher prevalence of comorbidities (48% vs. 26%; *p* = 0.039) and were significantly more likely to require invasive organ support during PICU admission, including mechanical ventilation (76% vs. 25%; *p* < 0.001) and vasopressor support (52% vs. 24%; *p* = 0.008) (Table S1). Survival outcomes according to baseline characteristics are presented in Table 4.

### 3.4. Predictors of Mortality

In the multivariable Cox proportional hazards model, prior therapeutic interventions before PICU admission and the requirement for mechanical ventilation remained independently associated with increased mortality (Table 5). Patients who had received therapeutic interventions prior to PICU admission had a more than threefold higher risk of death (HR: 3.19; 95% CI: 1.24–8.19). Similarly, the need for mechanical ventilation was associated with a significantly increased risk of mortality (HR: 3.02; 95% CI: 1.16–7.60). Although higher PRISM scores and the presence of comorbidities were more common among patients who died, these variables were not independently associated with mortality after adjustment for other covariates. No significant association was observed between tumor type and mortality in the adjusted analysis.

**Figure 2:**
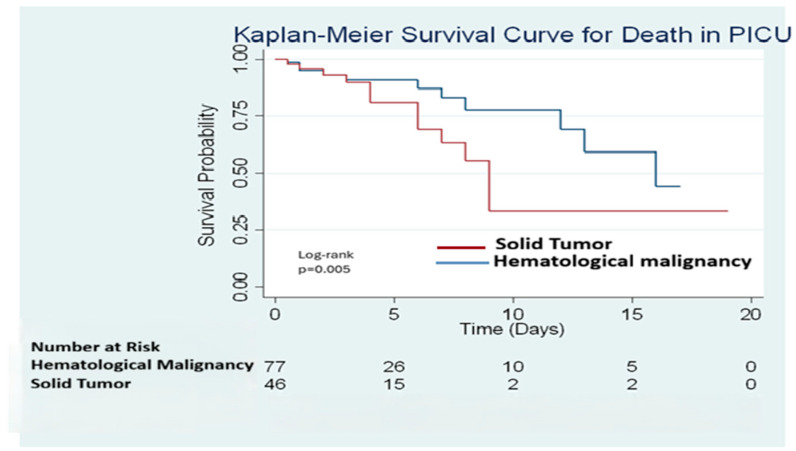
Kaplan–Meier survival curves comparing survival probability between pediatric oncology patients with hematologic malignancies and solid tumors admitted to the pediatric intensive care unit (PICU). Survival differed significantly between groups (log-rank test, *p* = 0.005).

Kaplan–Meier survival analysis demonstrated a statistically significant difference in survival between patients with hematologic malignancies and those with solid tumors (log-rank test, *p* = 0.005; Figure 2). However, tumor type did not remain an independent predictor of mortality after adjustment for clinical severity and critical care interventions in the multivariable Cox regression model.

## 4. Discussion

This study provides one of the most detailed evaluations to date of pediatric oncology patients requiring PICU admission in Saudi Arabia and adds important regional data to a literature largely derived from high-income Western healthcare systems. Our findings confirm that pediatric oncology patients admitted to the PICU represent a highly vulnerable population characterized by significant morbidity, frequent need for advanced organ support, and substantial mortality risk. In this cohort, the overall hospital mortality rate was 19%, with a PICU mortality of 10.3%, aligning with previously reported mortality rates ranging from 10 to 30% in critically ill pediatric oncology populations, depending on disease severity and indication for intensive care admission [3,7,10,13].

Consistent with prior international studies, sepsis and respiratory failure were the leading causes of critical illness and death in our cohort [3,6]. Sepsis frequently progressed to multi-organ failure, underscoring the profound vulnerability of immunocompromised children despite advances in antimicrobial therapy and supportive care [4,6,11]. The predominance of hematologic malignancies further reflects the heightened risk associated with prolonged neutropenia, intensive chemotherapy regimens, and cumulative immunosuppression [3,4,6]. Together, these findings emphasize the continued importance of early infection recognition, rapid antimicrobial escalation, and close respiratory monitoring within pediatric oncology care pathways.

The burden of organ dysfunction in this cohort was substantial, with nearly 40% of patients developing organ failure and more than one-third requiring mechanical ventilation or vasopressor support. These rates are comparable to those reported in large hemato-oncology PICU cohorts from Europe and North America [5,6,12]. Importantly, the need for invasive organ support has emerged as a strong marker of disease severity and a critical determinant of outcome. Mechanical ventilation, in particular, was independently associated with a threefold increase in mortality risk, consistent with multiple prior studies identifying respiratory failure as one of the strongest predictors of adverse outcomes in critically ill pediatric oncology patients [5,7,13,14,15]. These findings highlight the prognostic importance of respiratory compromise and suggest that strategies aimed at earlier recognition and escalation of care—before progression to advanced respiratory failure—may improve outcomes.

Notably, a substantial proportion of deaths occurred after discharge from the PICU, either during the same hospitalization or following readmission. This observation underscores the prolonged vulnerability of pediatric oncology patients beyond the acute phase of critical illness and highlights the importance of structured post-PICU surveillance, multidisciplinary follow-up, and early recognition of clinical deterioration. Similar patterns have been reported in previous studies, suggesting that survival beyond the PICU does not necessarily equate to recovery and may reflect ongoing physiologic instability [7,10,13].

Mortality in this cohort was driven primarily by clinical severity and treatment-related factors rather than malignancy subtype. Although earlier studies suggested worse outcomes among patients with hematologic malignancies [14], tumor type was not independently associated with mortality in our multivariable analysis. This likely reflects improvements in supportive care delivery, infection management, and standardized treatment protocols within specialized oncology centers. Nevertheless, infection—particularly sepsis—remained a dominant contributor to clinical deterioration, reinforcing the need for robust infection prevention strategies, early warning systems, and rapid-response pathways tailored to pediatric oncology patients.

Prior therapeutic exposure emerged as an independent predictor of mortality, conferring a threefold increased hazard of death. This finding highlights the cumulative physiologic burden of cancer-directed therapy and underscores the vulnerability of patients with extensive prior treatment exposure. Similar associations between cumulative treatment burden, organ toxicity, and adverse PICU outcomes have been reported previously [11,12,14]. These findings support the importance of close collaboration between oncology and critical care teams to identify high-risk patients and optimize the timing of PICU admission.

Although PRISM IV scores were higher among patients who died, they did not remain independently predictive of mortality in multivariable analysis. This finding aligns with prior reports questioning the discriminatory performance of general severity-of-illness scoring systems in pediatric oncology populations [10,12]. Factors such as chronic comorbidities, treatment-related toxicities, and disease- or therapy-associated immunosuppression may not be adequately captured by physiologic scoring systems alone, underscoring the need for oncology-specific prognostic tools that better reflect the complexity of this population.

By contextualizing PICU outcomes within the Saudi Arabian healthcare system, this study highlights both the progress achieved and the persistent challenges in pediatric oncology critical care. As pediatric oncology services continue to expand nationally, our findings emphasize the importance of early risk stratification, timely escalation of organ support, and standardized sepsis management protocols to reduce preventable mortality. Moreover, these data provide a valuable benchmark against which future quality improvement initiatives and multicenter collaborations can be evaluated.

Key limitations include the retrospective, single-center design and the potential for residual confounding. In addition, documentation regarding limitation of life-sustaining therapy and end-of-life decision-making was not consistently available, precluding detailed evaluation of these factors. Long-term post-PICU outcomes and functional recovery were also not systematically assessed. As such, the generalizability of these findings may be limited to similar tertiary referral settings. Future prospective, multi-center studies with extended follow-up are needed to refine prognostic indicators, validate severity scoring systems in pediatric oncology populations, and inform the development of standardized, evidence-based care pathways for critically ill children with cancer.

## 5. Conclusions

Pediatric oncology patients requiring PICU admission experience substantial morbidity and mortality, particularly those with prior therapeutic exposure or who require invasive organ support such as mechanical ventilation and vasopressor therapy. Sepsis and respiratory failure remain the primary drivers of critical illness in this population. Early recognition of clinical deterioration, aggressive infection prevention and management, and timely initiation of supportive therapies are essential to improving outcomes. Incorporation of validated severity scoring tools may aid in early risk stratification and support clinical decision-making. Multicenter, prospective studies with long-term follow-up are needed to further refine prognostic indicators and optimize care pathways for critically ill pediatric oncology patients.

## Figures and Tables

**Table 1:** Characteristics of pediatric oncology patients admitted to the Pediatric Intensive Care Unit (PICU).

Clinical Variables	Total *n* = 126
**Age, years (median [IQR])**	6 (3–10)
Age categories *	
<1 year	11 (8.7)
1–4 years	46 (36.5)
>4 years	69 (54.7)
**Gender**	
Male	51 (40.4)
Female	75 (59.5)
**Location Prior to PICU admission**	
Oncology ward	72 (57.1)
Emergency department	33 (26.1)
Others ****	21 (16.6)
**Presence of comorbidities**	
No	87 (69.0)
Yes	39 (30.9)
Associated comorbidity (*n* = 39) **	
Genetic disease	9 (7.1)
CNS disease	6 (4.7)
Renal disease	5 (3.9)
Gastrointestinal disease	1 (0.7)
Rheumatological disease	1 (0.7)
Immunological disease	3 (2.3)
Endocrine disease	2 (1.5)
Others ***	12 (9.5)
**Therapeutic interventions prior to PICU admission**	
No	37 (29.3)
Yes	89 (70.6)
**Type of treatment received (*n* = 89)**	
Chemotherapy alone	64 (71.9)
Chemotherapy + radiotherapy	7 (7.8)
Chemotherapy + radiotherapy + Surgery	5 (5.6)
Chemotherapy + Surgery	4 (4.4)
Chemotherapy + targeted therapy	3 (3.3)
Steroids	2 (2.2)
Others *****	4 (4.4)
**Reason for admission**	
Respiratory failure	27 (21.4)
Sepsis	52 (41.2)
Neurological deterioration	7 (5.5)
Renal impairment	2 (1.5)
Disease-related complication	10 (7.9)
Therapy-related complication ^	3 (2.3)
Disease progression	5 (3.9)
Others ******	20 (15.8)

* Age categories were defined a priori based on clinically relevant pediatric age groups commonly used in pediatric oncology and intensive care practice. ** Associated comorbidities included genetic disorders (e.g., trisomy 21, inherited immunodeficiency syndromes), central nervous system (CNS) disorders (e.g., epilepsy, developmental delay), chronic pulmonary disease (e.g., bronchial asthma, bronchopulmonary dysplasia), and cardiac disease (e.g., congenital heart disease). *** Refers to less frequent conditions not listed individually. **** Other areas including transfers from operating rooms or external facilities. ***** Other rare tumor not otherwise categorized. ****** Includes less frequent indications for PICU admission such as metabolic derangements, gastrointestinal complications, cardiovascular instability not requiring vasopressor support at admission, post–procedure monitoring not classified as postoperative care, and miscellaneous acute clinical deteriorations not captured in predefined categories. ^ Therapy-related complications included adverse events related to oncologic treatment, such as chemotherapy-induced neutropenic sepsis, treatment-related organ toxicity (including hepatic and renal dysfunction), and other acute complications directly attributable to cancer-directed therapy.

**Table 2:** Outcomes of pediatric oncology patients admitted to the Pediatric Intensive Care Unit.

		Diagnosis of Patients		
Outcome	Total (%)	Hematologic Malignancies 80 (63.4)	Solid Tumors 46 (36.5)	*p*-Value
**Survival status**				0.183
Alive	101 (80.1)	67 (83.7)	34 (73.9)	
Death	25 (19.8)	13 (16.2)	12 (26.1)	
**Non-survival**				0.848
During the PICU admission	13 (52.0)	7 (53.8)	6 (50.0)	
After the PICU admission	12 (48.0)	6 (46.1)	6 (50.0)	
Presence of organ failure during PICU admission **				0.778
No	76 (60.3)	49 (61.2)	27 (58.7)	
Yes	50 (39.6)	31 (38.7)	19 (41.3)	
Number of organs failing during PICU admission *^,^^				0.458
No organ failure	76 (61.0)			
One organ failure	34 (68.0)	20 (64.5)	14 (73.6)	
Multi-organ failure	15 (32.0)	10 (35.3)	5 (26.3)	
**Mechanical ventilation use**				0.168
No	81 (64.2)	55 (68.7)	26 (56.5)	
Yes	45 (35.7)	25 (31.2)	20 (43.4)	
**Duration if MV used in days (median [IQR])**	3 (2–7)	5 (2–11)	3 (1.5–4)	0.244
**Vasopressor support**				0.450
No	88 (69.8)	54 (67.5)	34 (73.9)	
Yes	38 (30.1)	26 (32.5)	12 (26.1)	
**Duration of vasopressor use, days (median [IQR])**	2 (2–4)	2 (1–3)	2 (2–5)	0.443
**Use of dialysis during PICU admission**				0.139
No	118 (93.6)	73 (91.2)	45 (97.8)	
Yes	8 (6.3)	7 (8.7)	1 (2.1)	
**Duration of dialysis use in days**	3 (2.5–7)	3 (2–7)	-	-
**Pediatric Risk Score for Mortality (PRISM) IV (median [IQR])**	15 (11–24)	15 (12–26)	15 (9–23)	0.146
**PICU length of stay in days (median [IQR])**	3 (2–7)	3 (2–7)	3 (2–7)	0.930

* Percentages were calculated among those with organ failure during PICU admission. ** Organ failure was defined as the need for active organ-supportive interventions (mechanical ventilation, vasopressor support, renal replacement therapy) or documented multiorgan failure during PICU admission. ^ Single-organ failure was defined as dysfunction of one organ system requiring active PICU-level support. Multi-organ failure was defined as concurrent dysfunction of two or more organ systems requiring organ-supportive interventions.

**Table 3:** Timing and Causes of Death Among PICU Patients (*n* = 25).

Timing of Death	Cause of Death	*n*
**During PICU stay (*n* = 13)**	Sepsis	10
	└─ Progression to multi-organ failure	5
	Respiratory failure	2
	Disease progression	1
**After PICU discharge—same admission (*n* = 4)**	Gastrointestinal bleeding	1
	Sepsis → multi-organ failure	1
	Respiratory failure	1
	Disease progression	1
**After discharge—subsequent admission (*n* = 8)**	Sepsis	4
	Disease progression	2
	Respiratory failure	2
**Total deaths**		**25 (19%)**

**Table 4:** Risk factors related to survival for pediatric oncology patients admitted to the pediatric intensive care unit (PICU).

Variables	Total *n* = 126	Survival		*p*-Value
		Alive *n* = 101	Death *n* = 25	
**Age**	6 (3–10)	7 (3–10)	3.7 (2.9–9)	0.285
**Gender**				0.957
Male	51 (40.4)	41 (40.5)	10 (40.0)	
Female	75 (59.5)	60 (59.4)	15 (60.0)	
**Presence of comorbidities**				0.039
No	87 (69.0)	74 (73.2)	13 (52.0)	
Yes	39 (30.9)	27 (26.7)	12 (48.0)	
**Therapeutic interventions prior to PICU**				0.511
No	37 (29.3)	31 (30.6)	6 (24.0)	
Yes	89 (70.6)	70 (69.3)	19 (76.0)	
Diagnosis of Patients *				0.183
Hematologic malignancies	80 (63.4)	67 (66.3)	13 (52.0)	
Solid tumors	46 (36.5)	34 (33.6)	12 (48.0)	
**Presence of organ failure during PICU admission**				0.063
No	76 (60.3)	65 (64.3)	11 (44.0)	
Yes	50 (39.6)	36 (35.6)	14 (56.0)	
**Mechanical ventilation use**				<0.001
No	81 (64.2)	75 (74.2)	6 (24.0)	
Yes	45 (35.7)	26 (25.7)	19 (76.0)	
**Vasopressor support**				0.008
No	88 (69.8)	76 (72.2)	12 (48.0)	
Yes	38 (30.1)	25 (24.7)	13 (52.0)	
**Length of stay in PICU, days**	3 (2–7)	3 (2–7)	6 (2–8)	0.25

* Diagnoses were grouped for analytic purposes according to the World Health Organization (WHO) classification of childhood cancers and do not represent clinical reclassification or disease progression.

**Table 5:** Bivariate Cox proportional hazards regression analysis to identify predictors of in-hospital mortality in the PICU.

Variables	HR (95% CI)	*p*-Value	HR (95% CI) *	*p*-Value
**Age**	0.99 (0.90–1.10)	0.977	-	
**Gender**				
Male	1			
Female	1.10 (0.49–2.47)	0.805		
**Presence of comorbidities**				
No	1			
Yes	2.10 (1.00–4.40)	0.048	1.95 (0.89–4.22)	0.091
**Therapeutic interventions prior to PICU**				
No	1			
Yes	2.70 (1.04–6.95)	0.039	3.19 (1.24–8.19)	0.016
**Diagnosis of Patients recategorized**				
Hematologic malignancies	1			
Solid tumors	2.15 (0.95–4.87)	0.066		
**Presence of organ failure during PICU admission**				
No	1			
Yes	1.27 (0.57–2.81)	0.554		
**Mechanical ventilation use**				
No	1			
Yes	3.00 (1.22–7.39)	0.016	2.97 (1.16–7.60)	0.023
**Vasopressor support use**				
No	1			
Yes	1.48 (0.66–3.30)	0.350		
**Pediatric Risk Score for Mortality (PRISM) IV**	1.04 (1.00–1.08)	0.022	1.02 (0.98–1.06)	0.262

* Adjusted for significant variables in univariate analysis.

## Data Availability

The datasets generated and analyzed during the current study are not publicly available due to patient privacy and institutional regulations but are available from the corresponding author on reasonable request and with appropriate ethical approvals.

## Supplementary Materials

The following supporting information can be downloaded at: https://www.mdpi.com/article/10.3390/children13010058/s1. Table S1: Survival status (during and after PICU admission) and study variables.

## Abbreviations

The following abbreviations are used in this manuscript:

ALL	Acute Lymphoblastic Leukemia
AML	Acute Myeloid Leukemia
CI	Confidence Interval
ED	Emergency Department
HLH	Hemophagocytic Lymphohistiocytosis
HR	Hazard Ratio
IQR	Interquartile Range
KASCH	King Abdullah Specialized Children’s Hospital
KM	Kaplan–Meier
NHL	Non-Hodgkin Lymphoma
PRISM	Pediatric Risk of Mortality
WHO	World Health Organization
