# Supplementary material for: Clinical Characteristics and Outcomes of Pediatric Oncology Patients Admitted to the Pediatric Intensive Care Unit: A Single Center Experience in Saudi Arabia
Source: Children (Basel). 2025 Dec 31;13(1):58. doi: 10.3390/children13010058 (PMC12840303; doi:10.3390/children13010058)
Supplement: Supplementary file 1 [file children-13-00058-s001.zip › children-4046720-supplementary.pdf]

Supplemental Table 1. Survival status (during and after PICU admission) and study variables

Variables	Nonsurvival during PICU N=13 (52%)	Nonsurvivable after PICU N=12 (48%)	p-value
**Age**	4 (3–9)	3.5 (1.9–6.5)	0.429
**Gender**			
Male	6 (46.1)	4 (33.3)	
Female	7 (53.8)	8 (66.6)	
**Presence of comorbidities**			
Yes	6 (46.1)	6 (50.0)	0.582
**Therapeutic interventions prior PICU**			
Yes	12 (92.3)	7 (58.3)	0.063
**Diagnosis of Patients_ recategorized**			
Hematologic Malignancies			
Solid tumors			
**Presence of organ failure during PICU admission**	10 (76.9)	4 (33.3)	0.036
Yes			
**Mechanical ventilation use**			
Yes	4 (66.6)	8 (42.1)	0.281
**Positive inotropic support**			
Yes	9 (69.2)	4 (33.3)	0.081
**PRISM**	19 (13–24)	23 (21–28)	0.120
